# Use of the Delphi method as an instrument of community participation in health needs assessment

**DOI:** 10.1057/s41271-025-00559-9

**Published:** 2025-04-29

**Authors:** Luna Rodríguez Pérez, Manuel Rodríguez Rodríguez, Rosario Vigo Ortega, Silvia Sicre Alonso, Tránsito Cebrián Valero, Pilar Mentuy Isus, Luis Gabriel Luque Romero

**Affiliations:** 1 Local Health Action Network of the Province of Seville, Disease Prevention, Health Promotion and Surveillance Unit, Health District of Seville, Seville, Spain; 2 QuirónSalud Sagrado Corazón Medical Center, Sistema Nacional de Salud (National Healthcare Network), Tomares, Seville, Spain; 3 Research Unit, Aljarafe-North Seville Health District, Servicio Andaluz de Salud, Calle Clara Jaime Melero, 2-4, 41008 Seville, Spain; 4 Disease Prevention, Health Promotion and Surveillance Unit, Aljarafe-North Seville Health District, Servicio Andaluz de Salud, Mairena del Aljarafe, Seville, Spain; 5 Local Health Action Network Section, Disease Prevention, Health Promotion and Surveillance Unit, Aljarafe-North Seville Health District, Servicio Andaluz de Salud, Mairena del Aljarafe, Seville, Spain; 6 Research Unit, Aljarafe-North Seville Health District, Servicio Andaluz de Salud, Seville, Spain; 7 Department of Preventive Medicine and Public Health, Faculty of Medicine, University of Seville, Seville, Spain; 8 Institute of Biomedicine of Seville (IBiS), Seville, Spain

**Keywords:** Health policy, Social determinants of health, Delphi technique, Social participation, Community participation

## Abstract

We conducted a comprehensive analysis of the usefulness of the Delphi technique for facilitating community participation in local health needs assessments within the Andalusian Local Health Action Network, Spain. We developed an ad hoc online questionnaire based on the Social Determinants of Health model and applied it to a panel of experts in two municipalities in the province of Seville (Andalusia, Spain) between May and June 2021. Our results reflected good panelist participation. The questionnaire successfully enabled the prioritization of both new and original items, some of which were incorporated into local health policies. We concluded that the Delphi method was effective for facilitating participation in local health needs assessments offering a replicable, cost-effective approach that accelerated local policy development and supported the implementation of Health in All Policies within local government.

## Key messages

- The Delphi Method (DM) is a cost-effective method for assessing and prioritizing health needs with community participation.
- The DM helps to define local health policies in dispersed or under-resourced areas.
- A Social Determinants questionnaire facilitates local implementation of Health in All Policies.

## Introduction

Since the release of the Ottawa Charter for Health Promotion in 1986, substantial evidence has emerged highlighting the critical role of social determinants in influencing health outcomes [1, 2]. As a result, intersectoral action has been increasingly promoted to improve the quality of life of the general population [3]. International [4], European [5], and Spanish [6] institutions have endorsed the Health in All Policies (HiAP) approach [4], which requires policymakers in all sectors to systematically consider the health impacts of their decisions [7]. Local governments play a crucial role in this framework, as they are the level of governance closest to the population and can implement policies that more accurately reflect local needs [8]. The development of HiAP at the local level is often based on health needs assessments (HNA), which rely on community participation [8] to ensure that policies resonate with citizens [10] and to design interventions aimed at improving health [9].

In our analysis, we explored the use of the Delphi method to facilitate community involvement in the HNA process. This initiative was conducted within the framework of the Local Health Action Network (RELAS), a project sponsored by the Andalusian Ministry of Health and Consumer Affairs to promote the implementation of HiAP by developing local health plans (LHPs) based on health needs assessment data [9], consisting of both quantitative data (demographic, epidemiological, economic, geographical, and social indicators) and qualitative insights gathered from the community through participatory methods such as focus groups, surveys, and interviews. A steering group then analyses this information to prioritize health needs and guide the development of interventions [9].

In the RELAS framework, this steering group, known as the Technical Advisory Group [11], comprises members from various health administration areas, with leadership provided by a provincial-level Expert Technical Advisor in health promotion. The Advisor supports and guides municipalities throughout the process.

During the COVID-19 pandemic in spring 2021, traditional participatory tools were impractical due to the need for face-to-face interaction. To prevent disruption of the process and ensure community input, the Health Promotion Team for the Aljarafe-North Seville Health District—composed of the Expert Technical Advisor, the Director of Disease Prevention, Health Promotion and Surveillance Unit, and leaders from the Local Health Action Network—opted to use the Delphi method.

We sought to analyze the implementation process and results of our experience with the Delphi methodology to promote intersectoral participation in the development of local health policies in two municipalities in the province of Seville, Andalusia.

## Data and methods

The Delphi technique is widely used in health sciences research to identify priorities and reach group consensus on key issues [12]. It is a structured method designed to gather expert opinions on a given topic [13]. In our analysis, we replicated key features of the Delphi method: purposive sampling, participant anonymity, iteration, and controlled feedback [14].

Fieldwork was conducted between May and June 2021 in two municipalities within the Aljarafe-North Seville Health District (Mairena del Alcor and Mairena del Aljarafe). After failing to find an existing instrument suited to our objectives, we designed an ad hoc online questionnaire based on Dahlgren and Whitehead’s (1991) framework of health determinants [14]. This framework is widely used due to its simplicity and practicality. The questionnaire, reviewed by experts and public representatives, was approved with only minor revisions.

The final version included 48 closed questions across six components: habits and lifestyle; education system; social environment; socioeconomic conditions; physical environment and neighborhood; and healthcare system. Participants rated the importance of each item on a 5-point Likert scale (from 0 = least important, to 5 = most important) and had the option to suggest new items relevant to their municipality.

In Rounds 1 and 2, participants rated the same items, along with new ones suggested after Round 1. The importance ratings from Round 1 were provided for reference, and the same criteria were used for re-rating. Each round lasted two weeks, with email reminders sent to non-respondents to ensure participation.

The Expert Technical Advisor processed the data and prepared a report on the results. The priorities identified through the Delphi process were added to the HNA and included in the local health plan, adopted by local councils to support the HiAP strategy.

The Health Promotion Team defined the selection criterion for experts as membership of the community, considering individuals who lived or worked in the municipality (geographical criterion) or belonged to a local sector or group (sector criterion). The selected expert profiles included representatives from citizens’ associations, key informants familiar with the municipality (for example, representatives of political groups, trade unions, and employers’ associations), municipal technical staff (from departments such as housing, environment, and public safety), staff from public institutions (schools, social services), primary care professionals (doctors, nurses, social workers), community pharmacists, and representatives of vulnerable groups.

The Health Promotion Team contacted potential participants by phone to invite them, explain the purpose of the analysis, and introduce the Delphi methodology. Individuals who demonstrated willingness, commitment, time availability, and communication skills [15] were included. Those who agreed to participate received an email from the Expert Technical Advisor with instructions, a link to the questionnaire, and the response deadline.

### Data analysis

We defined consensus in advance [16] and established two criteria: consistency of responses between rounds and the absence of new item contributions. To check consistency, we applied the Kruskal–Wallis test, comparing median scores for each item across rounds, with a significance level of *p* ≤ 0.05. To prioritize items, we used two conditions: a median score of 4 or 5 (indicating high importance) and an acceptable consensus level (relative interquartile range (RIR) ≤ 75%) [17]. Data from the online questionnaire were transferred to Open Office spreadsheets, and statistical analyses were performed using Epi Info 7 software [18].

## Results

Since our main objective was to analyze the usefulness of the Delphi method for facilitating community participation in local HNAs, we emphasize the application of the methodology. We also provide a brief analysis of the prioritized items as they relate to facilitating the development of local health policy.

There was a high level of participation relative to the initial number of people invited. The attrition rate between rounds was higher among male participants (Table 1). The distribution of items per health determinant varies by municipality, reflecting the addition of new items that correspond to specific local characteristics (Table 2). The distribution of prioritized items corresponds to the percentage of those included in the original questionnaire and those proposed by panelists (Table 3).

**Table 1:** Participant flows by sex and municipality

		Mairena del Alcor		Mairena del Aljarafe	
		Round 1	Round 2	Round 1	Round 2
Number invited		46 (100%)	35 (100%)	45 (100%)	28 (100%)
Number of participants		35 (76.0%)	25 (71.4%)	28 (62.2%)	26 (92.8%)
Sex					
	Female	31 (68.0%)	18 (70.0%)	26 (57.8%)	19 (73.3%)
	Male	15 (32.0%)	7 (30.0%)	19 (42.2%)	7 (26.7%)

**Table 2:** Evolution of items addressed in two Delphi rounds

	Mairena del Alcor				Mairena del Aljarafe			
Items grouped by SDH theme	Included in questionnaire	Proposed by panelists	No. prioritized of those included in the questionnaire	No. prioritized of those proposed	Included in questionnaire	Proposed by panelists	No. prioritized of those included in the questionnaire	No. prioritized of those proposed
Habits and lifestyles	7	2	3	0	7	4	5	1
Education system	9	8	0	0	9	6	0	0
Social environment	7	2	1	2	7	6	0	0
Socioeconomic conditions	6	3	5	1	6	3	6	1
Physical environment and neighborhood	13	3	0	0	13	15	0	3
Health system	6	3	2	2	6	6	2	6
Total	48	21	11	5	48	40	13	11

**Table 3:** Items included and prioritized after two Delphi rounds

	Items Round 1		Items Round 2			
	No. included in questionnaire	No. proposed by panelists	No. included in questionnaire	No. proposed by panelists	No. prioritized of those included in the questionnaire	No. prioritized of those proposed
Mairena del Alcor	48	21	69	0	11* (22.9%)	5* (23.8%)
Mairena del Aljarafe	48	40	88	0	13* (27.1%)	11* (27.5%)

*Only prioritized in Round 2

The steering group included some of the prioritized items (4 in Mairena del Alcor, 5 in Mairena del Aljarafe) in the LHPs, which the municipal councils then approved. Three prioritized problems associated with habits and lifestyle (poor diet, sedentary lifestyle, stress) were common to both municipalities. In the case of Mairena del Aljarafe, problems related to the health system were excluded from the LHPs and were referred to the management team of the reference Health District.

## Discussion

Our analysis aimed to examine the effectiveness of the Delphi method in promoting participation in Health Needs Assessments at the local level. It addresses a gap in the literature concerning the lack of concrete experiences with Health in All Policies, especially regarding implementation and practical recommendations [8, 19, 20], which limits local policy development within this paradigm [8]. The literature also emphasizes the need for research on facilitators and barriers to HiAP strategy development [8], particularly in the participation phase [19], a crucial element that influences policy decisions in the HiAP process [8, 20]. While focus groups and key informant interviews are commonly reported techniques, the use of the Delphi method for HNA is relatively new [9].

We found that the Delphi methodology was an efficient way to obtain a consensus list of SDHs in both municipalities. The literature supports the advantages of gathering information through this internet-based tool compared to face-to-face participatory techniques, highlighting its low cost and savings on typical expenses like participant travel and venue hire [21, 22]. Ravaghi et al. (2023) identified several challenges in conducting an HNA that Delphi can address [9]. First, Delphi is replicable and can engage the community without sacrificing methodological validity [23]. Second, the use of new technologies reduces logistical time and saves human and economic resources [16, 24]. We identified a potential barrier in the HNA process: local health professionals may lack skills in building consensus through participatory decision-making. To mitigate this, an external agent could administer the questionnaire to participants. Some authors have noted challenges in ensuring adequate participation and the risk of legitimizing pre-made decisions without allowing diverse citizen views to emerge [10]. The Delphi method can resolve this by facilitating participatory dialog over several rounds and allowing panelists to add their own opinions through open-ended questions.

In the RELAS project, we identified the following advantages. First, the Delphi method accelerates the participation phase compared to other methods, when conducting an HNA takes so long that it delays the preparation of the LHP [23]. Using this method, we reduced the overall process from an average of three years to one year. Second, with an external technical advisor facilitating the process, a single person can apply the tool in multiple communities. Third, it is possible to increase the number of panelists without a corresponding increase in complexity. Finally, the replicability of the technique allows us to identify changing needs within the same community and to compare different communities while identifying common factors influencing local needs. This method could play an important role in health areas where communities are scattered, where it is difficult for key informants to travel, or where the cost of human and economic resources is prohibitive.

We believe that community HNAs should focus on the social determinants of health [9]. Accordingly, we designed our initial questionnaire around Dahlgren and Whitehead’s model, which has been successfully used by practitioners and policymakers outside the health sector to conceptualize determinants [14]. Our questionnaire included a specific list of items for each determinant to encourage panelists to consider the broader context of social determinants of health when responding and to stimulate the suggestion of new items for each determinant. While the length of the questionnaire may contribute to participant attrition [25], there are no explicit criteria for defining an optimal number of items to prevent this. Following Lilly et al.’s recommendation, we grouped items into blocks [21]—in our case, by type of determinant—to help panelists maintain a determinants-based perspective of local reality and propose new items for each block [14].

We acknowledge that the criterion for inclusion as an expert panelist is controversial in the literature due to the challenge of measuring expertise. Therefore, it is necessary to define this criterion before forming the panel [13, 26]. Following this recommendation, we established community membership [9] as our criterion, using geography as the primary reference and representation of various sectors and local groups (health, education, the elderly, children, equality, etc.) as secondary. We considered panel members to be experts because they live or work in the municipalities and possess first-hand knowledge of the local realities. In the scientific literature, such individuals are referred to as affected parties, involved parties, or key informants in diagnostic processes who have knowledge of the situation under analysis regardless of title, status, or rank. They contrast with experts, who must have outstanding qualifications in the subject under study, such as academic credentials, special qualifications, or notable professional experience [27]. We set an initial minimum of 40 panelists, anticipating attrition in subsequent rounds, and thus ensured a diverse range of participant views in both municipalities. While there is no consensus on the optimal number of panelists, between 10 and 30 is generally considered sufficient [13, 20, 26].

After analyzing the prioritization results, we noted that the same three items related to lifestyle habits were prioritized and included in the LHPs of both municipalities [26]. This may be because these determinants are obvious and well known, suggesting there is still room to improve the dissemination of the Health in All Policies approach. Contributions made during the Delphi process could help the steering group identify new local realities requiring intervention.

The literature suggests that translating Health Needs Assessment findings into specific policies has been very limited. Ravaghi et al. (2023) conducted a scoping review of 169 studies: only five mentioned successful collaboration between the health sector and policymakers, and only four succeeded in setting or changing policy agendas [9], highlighting the lack of linkages between proposals resulting from participation and health policies [10]. The literature also mentions that political representatives often do not trust the results of citizen participation focused on social determinants of health [28]. In their 2023 review, Ling et al. discussed factors influencing the HiAP process in local governments and note the ambiguity and diversity of aspects affecting political decision-making, which may be one reason why some issues prioritized by citizens are not included in LHPs [28]. This was the case with items related to health services in Mairena del Aljarafe, which were excluded entirely.

### Study limitations

We identified most of the limitations in our analysis as relating to the panelists. First, because we did not stipulate a minimum number of representatives per sector but rather a total number, some sectors may have been over- or underrepresented, potentially affecting the number of prioritized items per determinant (more health sector panelists prioritizing more health-related issues). Second, we were unable to verify the level of expertise of the panelists. Since it is difficult to quantify community membership, we could not determine whether individuals living or working in the community were knowledgeable about it, especially if they had recently moved there [16]. Third, ensuring the independence of the panelists was challenging, as participation was limited to those with a pre-existing interest in the topic or a relationship with the project [25]. This relates to the difficulty of controlling for bias from panelists with personal or sectoral views of local realities [9]. Fourth, we did not directly check that the panelists understood the items included in the questionnaires. Differences in interpretation may limit consensus using this technique [16]. Conducting an online session via videoconference beforehand to explain the project and procedures could mitigate this limitation. Fifth, although we included local stakeholders, leaders, and key figures with a broad vision of various needs and social groups, we did not consider including individuals who lacked Internet access, had limited literacy, or belonged to particularly important disadvantaged groups in the locality. Furthermore, the initial validation of the questionnaire was a very simplified process due to the lack of a similar, ready-made tool. As a result, the questionnaire may have included items that were too general or open to different interpretations. We do not know whether the list of items prioritized by the panels would have been different if other techniques had been employed. Furthermore, we were unable to compare our results, even partially by sector, because there are no other similar local publications on these topics.

A common limitation of participatory processes is the ethical challenge of managing participants’ expectations about the ultimate impact of their contributions [9]. Although we made it clear throughout that Delphi prioritization was part of a broader process, we did not evaluate panelists’ perceptions of the process or the results.

### Future directions

With this analysis, we have paved the way for further development in designing and validating an initial questionnaire model that can be used to conduct Health Needs Assessments with a focus on Social Determinants of Health in local settings. Based on a simple, concrete, and accredited methodology, this tool would provide local governments with the opportunity to implement Health in All Policies and to assess whether channeling citizen participation in the HNA process through a specific validated tool enables policymakers to feel more comfortable implementing HiAP at the local level.

Another avenue for future research with the same objective is the evaluation and validation of panels specifically for the use of the Delphi method in local settings. While there are recommendations in the literature for creating and energizing expert panels, most are at the scientific level, typically focusing on assessing expertise on a single topic to be analyzed. Expert assessment may mean expertise in the local area by virtue of living and working there (geographic criterion) and expertise in a specific determinant in that locality (sector criterion). Therefore, it is crucial to establish specific criteria for panel composition and to assess the extent to which uneven representation of different sectors within a panel influences the results obtained.

Another future line is to analyze experts’ expectations and satisfaction with the method and the tool, as this may help identify possible biases and causes of attrition during the process.

## Conclusions

In this Viewpoint, we presented the mechanism and process used to produce a health needs assessment and its degree of implementation in a local health plan approved by local government, but the medium- to long-term development and impact of this policy remain to be evaluated. We consider the Delphi method to be an effective and cost-efficient strategy for involving citizens in the development of local health policies, particularly in highly geographically dispersed areas or when accessibility is limited, such as during confinement phases of the COVID-19 pandemic.

## Data Availability

Our Institution's data protection policy does not allow us to provide open data, except in those situations in which it is previously authorized.

## References

[1] Wilkinson R, Marmot M. Los hechos probados (Second edition): Los determinantes sociales de la Salud [Internet]. 2nd ed. Secretaría General Técnica, editor. Organización Mundial de la Salud. Madrid, España: Ministerio de Sanidad y Consumo; 2006. https://www.sanidad.gob.es/areas/promocionPrevencion/promoSaludEquidad/equidadYDesigualdad/docs/hechosProbados.pdf. Accessed 4 May 2023.

[2] Organización Mundial de la Salud. Subsanar las desigualdades en una generación. Alcanzar la equidad sanitaria actuando sobre los determinantes sociales de la salud [Internet]. Comisión sobre Determinantes Sociales de la Salud. 2009. https://apps.who.int/iris/handle/10665/44084. Accessed 4 May 2023.

[3] Artazcoz L, Oliva J, Escribà-Agüir V, Zurriaga O. La salud en todas las políticas, un reto para la salud pública en España. Informe SESPAS 2010. Gac Sanit. 2010;24 Suppl 1:1–6. Erratum in: Gac Sanit. 2011 May-Jun;25(3):260. 10.1016/j.gaceta.2010.10.006. https://www-clinicalkey-es.bvsspa.idm.oclc.org/#!/content/playContent/1-s2.0-S0213911110002633?returnurl=https:%2F%2Flinkinghub.elsevier.com%2Fretrieve%2Fpii%2FS0213911110002633%3Fshowall%3Dtrue&referrer=https:%2F%2Fpubmed.ncbi.nlm.nih.gov%2F. 10.1016/j.gaceta.2010.10.006 21075491

[4] Hadar J, Bank W. Promoting Health in All Policies and intersectoral action capacities [Internet]. World Health Organization. 2022. https://www.who.int/activities/promoting-health-in-all-policies-and-intersectoral-action-capacities. Accessed 4 May 2023.

[5] Ollila E, Ståhl T, Wismar M, Lahtinen E. Health in all Policies in the European Union and its member states [Internet]. 2006. https://ec.europa.eu/health/ph_projects/2005/action1/docs/2005_1_18_frep_a4_en.pdf. Accessed 4 May 2023.

[6] Ministerio de Sanidad. Estrategia de salud pública 2022. ESP 2022 [Internet]. Madrid, España: Ministerio de Sanidad; 2022. https://www.sanidad.gob.es/ciudadanos/pdf/Estrategia_de_Salud_Digital_del_SNS.pdf. Accessed 4 May 2023.

[7] World Health Organization. Helsinki statement framework for country action. Minist Soc Aff Heal Finl. 2014;978:92–4.

[8] Van Vliet-Brown CE, Shahram S, Oelke ND. Health in All Policies utilization by municipal governments: scoping review. Health Promot Int. 2017;33(4):713–22. 10.1093/heapro/dax008. 10.1093/heapro/dax008 28334905

[9] Ravaghi H, Guisset AL, Elfeky S, Nasir N, Khani S, Ahmadnezhad E, Abdi Z. A scoping review of community health needs and assets assessment: concepts, rationale, tools and uses. BMC Health Serv Res. 2023;23(1):44. 10.1186/s12913-022-08983-3. 36650529 10.1186/s12913-022-08983-3 PMC9847055

[10] Mira JJ, Carrillo I, Navarro I, Guilabert M, Vitaller J, Pérez-Jover V, Aguado H. La participación ciudadana en salud. Revisión de revisiones. An Sist Sanit Navar. 2018;41(1):91–106. 10.23938/ASSN.0172. 29465091 10.23938/ASSN.0172

[11] Ruiz Fernández J, Carlos J, Camarillo R, Reyes L, Francisco A, Benítez R, et al. Manual para la elaboración de planes locales de salud. 2017. https://www.redlocalsalud.es/wp-content/uploads/2017/03/Planes_Locales_de_Salud2_indice_final.pdf. Accessed 4 May 2023.

[12] Shang Z. Use of Delphi in health sciences research: a narrative review. Medicine. 2023;102(7):e32829. 10.1097/MD.0000000000032829. 36800594 10.1097/MD.0000000000032829 PMC9936053

[13] Masdeu Ávila C. The Delphi method in health. Hipertens Riesgo Vasc. 2015;32(Suppl. 1):12–6. 10.1016/S1889-1837(15)30003-9.26179853

[14] Reguant-Álvarez M, Torrado-Fonseca M. El método Delphi. REIRE, Revista d’Innovació i Recerca en Educació. 2016;9(1):87–102. 10.1344/reire2016.9.1916.

[15] Dahlgren G, Whitehead M. The Dahlgren-Whitehead model of health determinants: 30 years on and still chasing rainbows. Public Health. 2021;199:20–4. 10.1016/j.puhe.2021.08.009. 34534885 10.1016/j.puhe.2021.08.009

[16] Von Der Gracht HA. Consensus measurement in Delphi studies: review and implications for future quality assurance. Technol Forecast Soc Change. 2012;79(8):1525–36. 10.1016/j.techfore.2012.04.013.

[17] Foth T, Efstathiou N, Vanderspank-Wright B, Ufholz LA, Dütthorn N, Zimansky M, Humphrey-Murto S. The use of Delphi and Nominal Group Technique in nursing education: A review. Int J Nurs Stud [Internet]. 2016 [cited 2023 May 3];60:112–20. 10.1016/j.ijnurstu.2016.04.015. Available from: https://www-sciencedirect-com.bvsspa.idm.oclc.org/science/article/pii/S0020748916300359?via%3Dihub. 10.1016/j.ijnurstu.2016.04.015 27297373

[18] CDC. Epi InfoTM, Division of Health Informatics & Surveillance (DHIS), Center for Surveillance, Epidemiology & Laboratory Services (CSELS). 2021. Windows | Epi InfoTM | CDC. https://www.cdc.gov/epiinfo/pc.html.

[19] Nasa P, Jain R, Juneja D. Delphi methodology in healthcare research: How to decide its appropriateness. World J Methodol. 2021;11(4):116–29. 10.5662/wjm.v11.i4.116. 34322364 10.5662/wjm.v11.i4.116 PMC8299905

[20] Quilling E, Kuchler M, Tollmann P, Osterhoff A, Leimann J. Needs to create healthy living environments-a two-stage Delphi survey in europe to identify facilitating factors and barriers in municipal health promotion. Int J Environ Res Public Health. 2022;19(9):5084. 10.3390/ijerph19095084. 35564480 10.3390/ijerph19095084 PMC9105741

[21] Lilly K, Kean B, Hallett J, Robinson S, Selvey LA. Factors of the policy process influencing Health in All Policies in local government: a scoping review. Front Public Health. 2023;11:1010335. 10.3389/fpubh.2023.1010335. 36844855 10.3389/fpubh.2023.1010335 PMC9949293

[22] Romero-Collado A. Essential elements to elaborate a study with the (e)Delphi method. Enferm Intensiva (Engl Ed). 2021;32(2):100–4. 10.1016/j.enfie.2020.09.003. 10.1016/j.enfie.2020.09.003 PMC9750506 34099261

[23] Baumann LA, Brütt AL. Comparing an in-person workshop and a postal Delphi Survey for involving health service users in health care and health research prioritization. Health Expect. 2023;26(1):199–212. 10.1111/hex.13646. 36346143 10.1111/hex.13646 PMC9854299

[24] Akins RB, Tolson H, Cole BR. Stability of response characteristics of a Delphi panel: application of bootstrap data expansion. BMC Med Res Methodol. 2005;5:37. 10.1186/1471-2288-5-37. 16321161 10.1186/1471-2288-5-37 PMC1318466

[25] Escuela Andaluza de Salud Pública. II ENCUENTRO DE LAS REDES DE ACCIÓN LOCAL EN SALUD. 2014. https://www.redlocalsalud.es/wp-content/uploads/2020/10/500704-Programa-Encuentro-RELAS2014-DEF_revisado-1-noviembre.pdf.

[26] Savic LC, Smith AF. How to conduct a Delphi consensus process. Anaesthesia. 2023;78(2):247–50. 10.1111/anae.15808. 35816561 10.1111/anae.15808

[27] Niederberger M, Spranger J. Delphi technique in health sciences: a map. Front Public Health. 2020;8:457. 10.3389/fpubh.2020.00457. 33072683 10.3389/fpubh.2020.00457 PMC7536299

[28] Guglielmin M, Muntaner C, O’Campo P, Shankardass K. A scoping review of the implementation of health in all policies at the local level. Health Policy. 2018;122(3):284–92. 10.1016/j.healthpol.2017.12.005. 29305241 10.1016/j.healthpol.2017.12.005

